# The Effect of Speech Masking on the Human Subcortical Response to Continuous Speech

**DOI:** 10.1523/ENEURO.0561-24.2025

**Published:** 2025-04-01

**Authors:** Melissa J. Polonenko, Ross K. Maddox

**Affiliations:** ^1^Department of Speech-Language-Hearing Sciences, University of Minnesota, Minneapolis, Minnesota, 55455; ^2^Departments of Biomedical Engineering and Neuroscience, University of Rochester, Rochester, New York, 14627; ^3^Kresge Hearing Research Institute, Department of Otolaryngology – Head and Neck Surgery, University of Michigan, Ann Arbor, Michigan, 48109

**Keywords:** auditory brainstem response, EEG, masking, speech, temporal response function

## Abstract

Auditory masking—the interference of the encoding and processing of an acoustic stimulus imposed by one or more competing stimuli—is nearly omnipresent in daily life and presents a critical barrier to many listeners, including people with hearing loss, users of hearing aids and cochlear implants, and people with auditory processing disorders. The perceptual aspects of masking have been actively studied for several decades, and particular emphasis has been placed on masking of speech by other speech sounds. The neural effects of such masking, especially at the subcortical level, have been much less studied, in large part due to the technical limitations of making such measurements. Recent work has allowed estimation of the auditory brainstem response (ABR), whose characteristic waves are linked to specific subcortical areas, to naturalistic speech. In this study, we used those techniques to measure the encoding of speech stimuli that were masked by one or more simultaneous other speech stimuli. We presented listeners with simultaneous speech from one, two, three, or five simultaneous talkers, corresponding to a range of signal-to-noise ratios (clean, 0, −3, and −6 dB), and derived the ABR to each talker in the mixture. Each talker in a mixture was treated in turn as a target sound masked by other talkers, making the response quicker to acquire. We found consistently across listeners that ABR Wave V amplitudes decreased and latencies increased as the number of competing talkers increased.

## Graphical abstract

**Figure:**
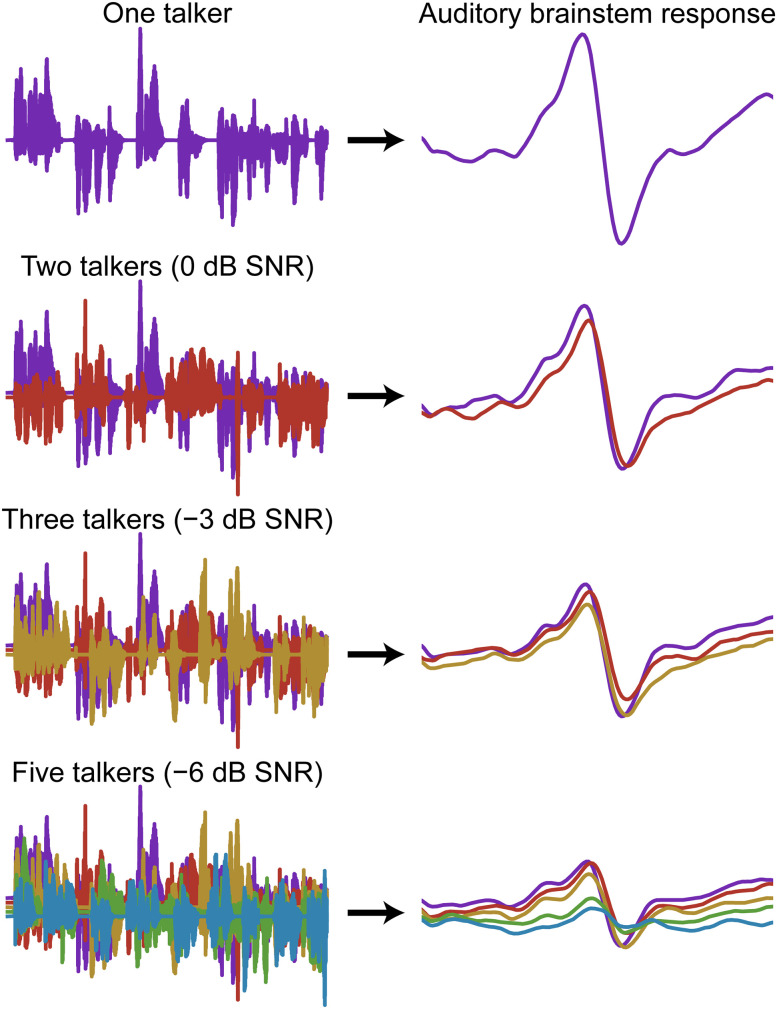
Visual Abstract

## Significance Statement

Trying to listen to someone speak in a noisy setting is a common challenge for most people, due to auditory masking. Masking has been studied extensively at the behavioral level, and more recently in the cortex using EEG and other neurophysiological methods. Much less is known, however, about how masking affects speech encoding in the subcortical auditory system. Here we presented listeners with mixtures of simultaneous speech streams ranging from one to five talkers. We used recently developed tools for measuring subcortical speech encoding to determine how the encoding of each speech stream was impacted by the masker speech. We show that the subcortical response to masked speech becomes smaller and increasingly delayed as the masking becomes more severe.

## Introduction

Speech is foundational to communication for hearing people. While some speech is heard under quiet conditions like a living room or office, noisy listening conditions like busy streets, crowded restaurants, or transit are daily scenarios that make understanding speech difficult. Masking is the phenomenon by which this noise negatively impacts the processing of speech. Overcoming masking to understand speech is built on an expansive neurophysiological network that begins in the cochlea and involves numerous interconnected regions in the brainstem, thalamus, and cortex. While many studies have investigated masking, little is known about the neural encoding of masked, naturally uttered speech in human listeners, especially in the earlier subcortical stages of the auditory system. The present study was aimed at addressing that gap.

Masking of speech has been extensively studied through psychophysical means for the better part of a century (Licklider, 1948; Miller and Licklider, 1950). These behavioral studies have described the perceptual phenomena of masking in fine detail, including distinct (but typically co-occurring) types of masking, such as energetic and informational masking (Brungart et al., 2001). There are also well known attributes of stimuli or listening scenarios that offer some release from masking, such as spatial separation (Freyman et al., 2001) or the addition of visual cues (Sumby and Pollack, 1954). Masking’s effects are often worsened in people with hearing loss and those using hearing aids and cochlear implants (Working Group on Speech Understanding and Aging, 1988; Fu and Nogaki, 2005), making its study clinically important as well. In some specific instances, behavioral masking effects can be closely tied to the underlying physiology, such as tone-in-noise paradigms designed to assess cochlear tuning in humans (Oxenham and Shera, 2003). But generally speaking, it is difficult to infer masking’s specific neurophysiological effects from behavioral data (which by their nature depend on the entire auditory system), especially for natural speech stimuli.

Neural studies of masking are less common. In humans, cortical responses to masked natural speech have been studied to better understand selective auditory attention (Mesgarani and Chang, 2012; Ding and Simon, 2013; O’Sullivan et al., 2014). Studies aimed at subcortical effects of speech masking have used repeated single syllables in multitalker babble noise (Song et al., 2011). There has also been animal work using conspecific stimuli to investigate aspects of masking, such as the masker type (Narayan et al., 2007) and spatial separation (Maddox et al., 2012), but these are harder to link to human perception.

There is a clear need for linking our understanding of the neural and perceptual effects of masked speech. Hearing aids can restore audibility for people with hearing loss, but speech understanding, especially in noise, remains a challenge for many (Davidson et al., 2022). Identifying the underlying neural causes may help us understand some of the variability across listeners with similar hearing loss configurations and may also aid in developing and fitting better hearing aid signal processing algorithms. Even people with clinically normal hearing thresholds can have significant difficulty understanding speech under noisy conditions. Multiple hypotheses exist regarding the neural and cochlear causes of such listening challenges (Bharadwaj et al., 2014; Carney, 2018), but a physiological test that accurately predicts speech-in-noise perception in humans with normal audiograms has been elusive (Prendergast et al., 2015). Many of these attempts have comprised responses to transient stimuli (e.g., clicks, tone bursts, repeated syllables), sometimes in the presence of masking noise, which bear little resemblance to natural speech.

The goal of this paper was to better understand masking’s effects on the subcortical neural encoding of naturally uttered speech in human listeners. To do this, we leveraged our recently developed method for determining the auditory brainstem response (ABR) to speech (Polonenko and Maddox, 2021a), which is built on the temporal response function (TRF) framework (Lalor and Foxe, 2010). Whereas our previous work was aimed at encoding of single talkers, here we determined the ABR (as a TRF reflecting subcortical response component) to speech in quiet as well as in the presence of varying numbers of other talkers. We found robust trends in the latency and amplitude of the responses as the number of talkers (and thus level of masking) increased. We also assessed how quickly those masking effects could be ascertained in individual listeners, which is critical for any measure that is a potential candidate for clinical use.

## Materials and Methods

### Human participants

All experimental methods were approved by the University of Rochester Research Subjects Review Board. Participants gave informed consent before participating, were paid for their time, and had confirmed hearing thresholds of 20 dB HL or better at octave frequencies from 500 to 8,000 Hz in both ears. Twenty-five participants were recruited with ages from 19 to 37 years (mean ± SD of 23.4 ± 5.5 years) with 16 identifying as female and 9 as male.

### Behavioral task

While peaky speech preserves natural speech’s spectrotemporal structure and subjectively sounds very similar, it was important to confirm that masking effects were also similar (see next section for details of peaky speech construction). We conducted a listening task with speech-on-speech masking so that we could compare the speech reception thresholds of natural and peaky speech.

We used the standard coordinate response measure (CRM) sentences as target speech (Brungart, 2001), in a task based on Gallun et al. (2013). Sentences were randomly chosen from any of the male talkers and had the call sign “Charlie.” The targets were presented at 65 dB SPL over Etymotic ER-2 earphones. The masker sounds were speech randomly selected from 150 ten-second segments from the five stories used as stimuli in the EEG task (described in the next section). They were then shortened to be the same length as the CRM sentence. Each masker was a combination of segments from three distinct talkers from the set of five added together. The sound level was limited to 80 dB SPL, which meant that the minimum (most difficult) target-to-masker ratio (TMR) tested was −14.5 dB.

Before testing, participants were trained to make sure they understood the task. This was accomplished by presenting target sentences with no masker. After each response, a binomial test was performed on the responses so far under the null assumption that the responses were random. Each participant moved on to the main task once that assumption could be rejected at *p *= 0.05 for both natural and peaky speech. All participants passed training easily, in 3–8 trials per stimulus type.

Speech reception thresholds for the natural and peaky speech targets were determined through an adaptive track in which the masker level was varied. There was a random wait of 1–4 s between trials. The track started with five up–one down for three reversals and then became a one up–one down track for eight reversals. Those latter eight were averaged to estimate the 50% correct point, where a correct trial was defined as choosing the correct color and number combination. With four color choices and eight numbers, the chance level was 1 in 32 or 3.1%.

Within each of six blocks, an adaptive track was run for each stimulus type. The final threshold for each stimulus type was taken as the average of the thresholds from the six blocks. Trials from the tracks were interleaved randomly, but the tracks were updated separately. We also ensured that one tracker never got more than two trials ahead of the other. Four breaks of a minimum of 15 s were forced during the experiment, and participants were allowed to take other breaks *ad libitum*, including extending the forced breaks.

### EEG

#### Stimuli

**Figure 1.**
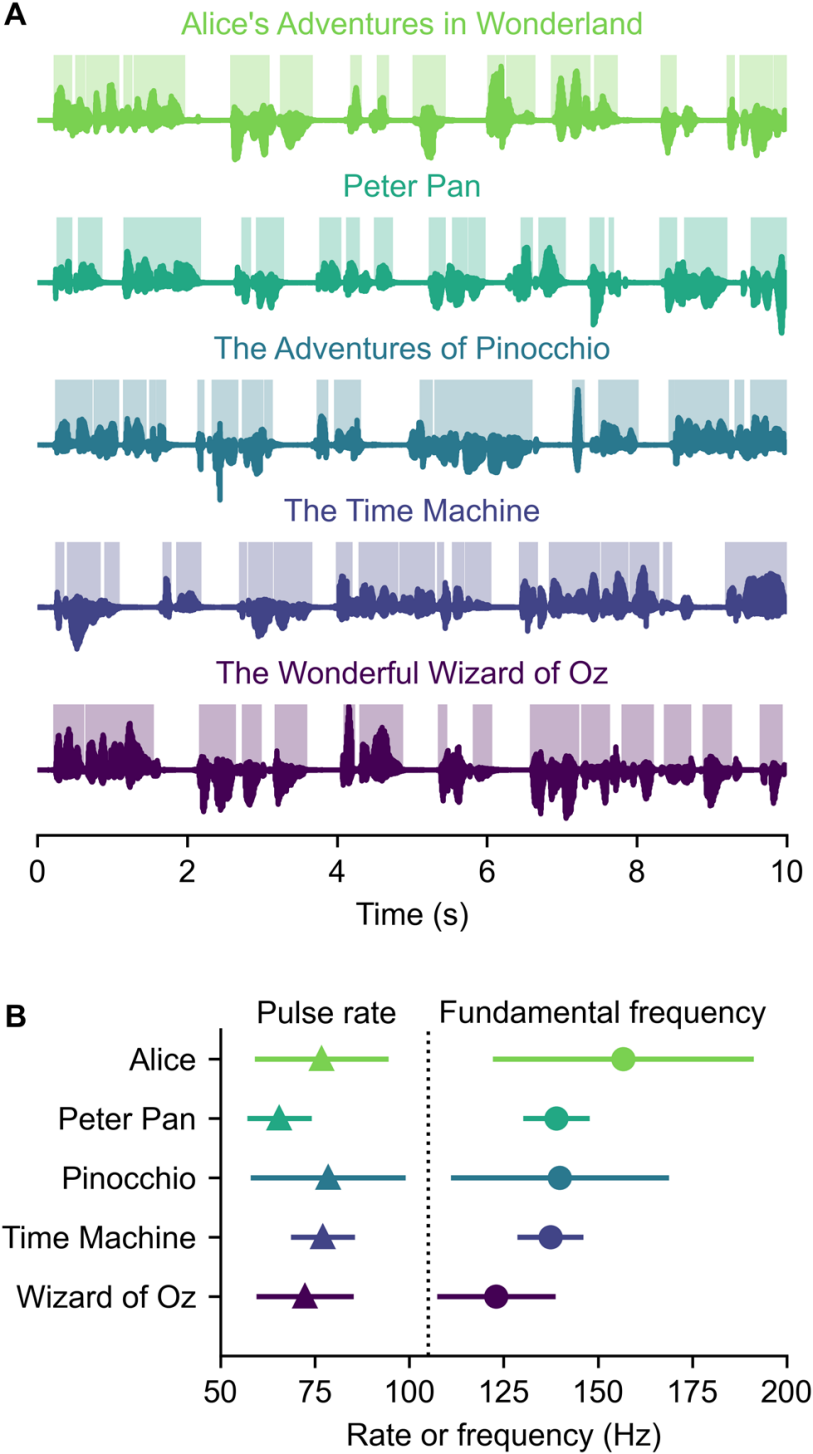
Experimental stimuli. ***A***, An example of 10 s from each of the five audiobooks. Dark lines show waveforms, with paler highlighted regions corresponding to voiced portions of speech (i.e., where there were glottal pulses). ***B***, The overall glottal pulse rate (number of pulses per second) and average fundamental frequency (number of pulses per second of voiced speech) of each audiobook.

Five audiobooks, read by five different narrators, were downloaded from the open source LibriVox Collection: *Alice*’*s Adventures in Wonderland* (Carroll, 2020), *Peter Pan* (Barrie, 2017), *The Adventures of Pinocchio* (Collodi, 2012), *The Time Machine* (Wells, 2011), and *The Wonderful Wizard of Oz* (Baum, 2007). All narrators were male, since previous work has shown that peaky speech narrated by folks with lower f0s yields larger responses (Polonenko and Maddox, 2021a, 2024). Figure 1 shows a 10 s sample of each talker and their pulse rates and fundamental frequencies. Each story was filtered so that its long-term power spectrum matched the average spectrum of all five stories (Shan et al., 2024). Responses were then converted to peaky speech in the same manner as prior work (Polonenko and Maddox, 2021a). Briefly, converting natural speech to peaky speech involves the following steps:

1. Identify the times of glottal pulses (present during vowels and voiced consonants) using Praat (Boersma and Weenink, 2024) controlled in Python with Parselmouth (Jadoul et al., 2018).
2. Create a dynamic cosine whose phase advances by 2*π* between each glottal pulse, such that its instantaneous frequency matches that of the speech's fundamental frequency. The amplitude of the cosine is determined from the spectrogram of the original speech.
3. Repeat Step 2 for all harmonics (integer multiples of the fundamental frequency), with the exception that the frequency of each cosine is 2π*k* for the *k*th harmonic. Add the fundamental and all harmonics together.
4. Combine the synthesized speech (which contains only the voiced portions of the speech) with the unvoiced portions of the original speech.

We created the different signal-to-noise ratio (SNR) conditions by mixing talkers from different stories together, with each talker always at the same overall level as the others. By mixing two talkers together, each talker was masked by one other talker at the same level, so the SNR was 0 dB (signal and noise were the same). For three talkers, one talker was masked by two others (doubling the noise energy), so the SNR was −3 dB. An SNR of −6 dB was created from five talkers (each one masked by four others). In general, the SNR could be calculated as $-10\log_{10}\;(N-1)$, where *N* was the number of talkers. Single talkers were also presented in isolation, with that condition labeled as “Clean.” Combinations of narrators were created so that each story was presented with equal frequency over the course of the experiment. Each talker was presented diotically at 65 dB SPL, meaning that with five talkers the level was 72 dB SPL. This was deemed in piloting to be the highest level that participants considered comfortable. Trials from each SNR condition were recorded for 30 min, meaning there were 6, 12, 18, and 30 min of each story presented for the clean and 0, −3, and −6 dB SNR conditions.

An advantage of our speech presentation paradigm is that the response to all talkers in a mixture could be calculated separately and then averaged. By doing so, even though only 30 min of data were recorded from each SNR condition, there were 30, 60, 90, and 150 min of total speech presented for the clean and 0, −3, and −6 dB SNR conditions. This effective increase in recording time as SNR decreased led to reduced noise in the resulting waveforms, largely ameliorating the problem of smaller amplitude responses in those conditions (see Results).

Stimuli were presented in 10 s epochs with order randomized. Experiments were controlled with a Python script built on the expyfun module (Larson et al., 2014). Stimuli were played using an RME Babyface Pro sound card at 48,000 samples/s and presented over Etymotic Research ER-2 tube earphones.

#### Recording

Participants were seated in reclining chair in a darkened sound-treated room. They were given the option of resting or watching silent, subtitled video. They could take a break at any time by pressing a button to pause the experiment.

EEG were recorded using the BrainVision ActiCHAMP amplifier and two EP-Preamp ABR preamplifiers. Two bipolar channels were recorded: FCz referenced to each of the earlobes. The ground electrode was placed on the forehead at Fpz. The two channels were averaged during preprocessing to increase SNR and because stimuli were presented diotically.

Triggers for synchronizing stimulus presentation to EEG data were generated using the sound card’s optical digital audio output, converted to TTL voltages using a custom-made trigger box (Maddox, 2020), and then fed to the EEG amplifier. The 0.9 ms earphone tube delay was corrected in preprocessing. Triggers were also used to precisely mark the end of each trial.

### Preprocessing and ABR TRF calculation

Preprocessing and analysis were done in python scripts that made heavy use of the mne package (Gramfort et al., 2013; Larson et al., 2024). Raw data were high- and low-pass filtered at 1 and 2,000 Hz using first-order causal infinite impulse response (IIR) filters and then notch filtered at odd multiples of 60 Hz, also with causal IIR filters, to remove power line noise. EEG epochs were created for each trial that began 1 s before stimulus start and ended 1 s after stimulus end. ABR TRFs were calculated from each epoch through frequency-domain deconvolution, using the glottal pulse trains as the stimulus regressor, exactly as in Polonenko and Maddox (2021a). ABRs for each participant were calculated for each talker in each epoch and then averaged across talkers and epochs to yield a single response for each SNR condition (see Extended Data Fig. 3-1 for separate responses to each talker). To improve signal quality, each trial’s contribution to the average was weighted by the inverse of the EEG variance during that period, a technique we have shown to be effective in several past studies (Polonenko and Maddox, 2019, 2021a,b). After ABRs were calculated, they were bandpass filtered between 150 and 2,000 Hz (causal, first-order, IIR) for better visualization of early ABR waves.

10.1523/ENEURO.0561-24.2025.f3-1 Figure 3-1 Grand average waveforms in response to each of the five stories. Plotted as in Figure 3. Download Figure 3-1, TIF file.

For experiments with longer stimuli, drift between the sound card and EEG clocks can cause smearing of responses. We eliminated this problem by using the triggers that marked the start and end of each trial to calculate the drift and compensate by resampling the glottal pulse train regressor before deconvolution.

### ABR analysis

ABR Wave V peaks and troughs were picked automatically by taking the maximum and minimum point of the waveform between 6.8 and 11.5 ms. Before picking, each waveform was low-pass filtered at 500 Hz to reduce noise using a zero-phase filter so that peaks did not shift. All picks were inspected by both authors to confirm validity. Individual participant waveforms for each SNR with labeled peaks can be seen in Extended Data Figure 3-2. The Wave V amplitude was taken as the difference between the peak and trough voltages. The Wave V latency was taken as the time of the peak voltage.

**Figure 2.**
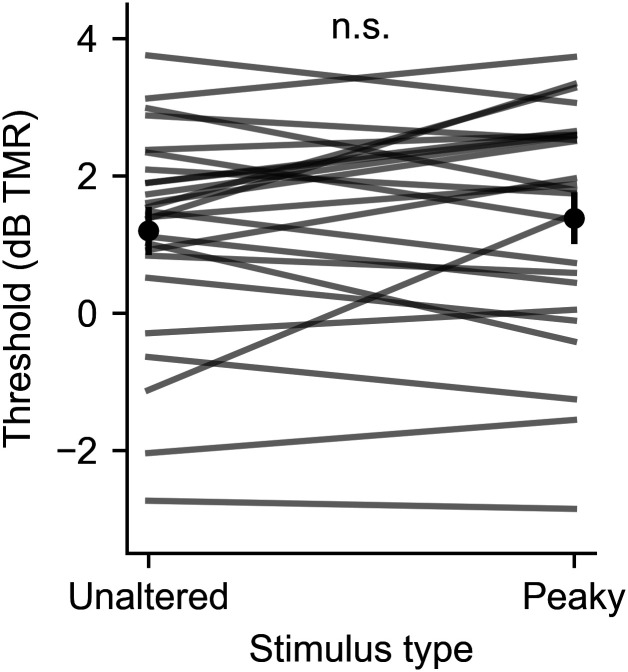
Comparison of speech reception thresholds for unaltered speech versus resynthesized peaky speech. Gray lines show individual participants. Black points and error bars show mean and ±1 SEM.

**Figure 3.**
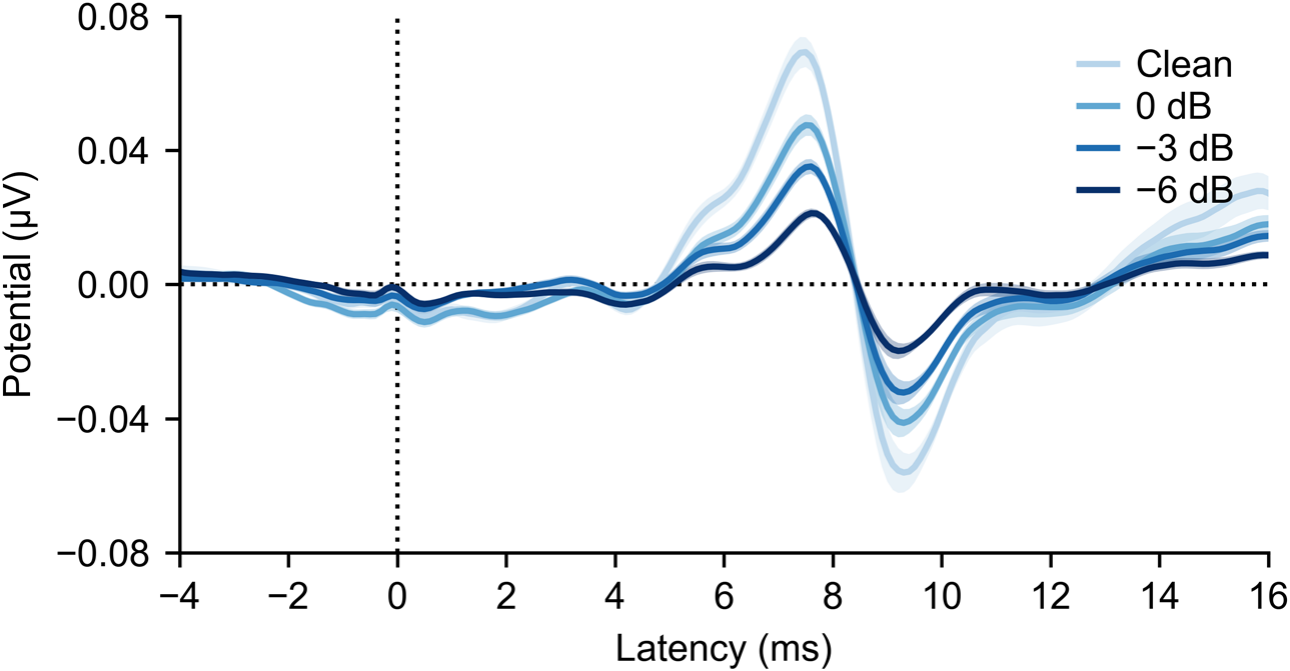
Grand average waveforms across 25 participants for each SNR (darker colors indicate lower SNR). Wave V is the prominent peak at ∼7.5 ms. Shaded areas show ±1 SEM around the mean.

10.1523/ENEURO.0561-24.2025.f3-2 Figure 3-2 Individual participants’ responses for each SNR condition. Wave V peaks and troughs are marked with dots and exes, respectively. Download Figure 3-2, TIF file.

We assessed waveform quality by computing the waveform SNR, not to be confused with stimulus SNR. The signal + noise variance, $\sigma_{S+N}^{2}$, was computed from the response between 4 and 12 ms (a segment duration of 8 ms). The noise variance, $\sigma_{N}^{2}$, was computed by dividing the prestimulus period from the same waveform (which contained only noise) into adjacent 8 ms segments, computing the variance of each, and averaging. The SNR in decibels could then be calculated as follows:

$$SNR\;=\;10\log_{10}\frac{\sigma_{S+N}^{2}-\sigma_{N}^{2}}{\sigma_{N}^{2}}.$$

Wave V amplitude and latency changes with stimulus SNR were evaluated with a linear mixed effects model with fixed effects of SNR, calculated as the logarithm of the number of talkers in the mixture to transform the data for linearity, gender, and its interaction with SNR and random effects of intercept and slope per participant. The coefficients for each participant were extracted from the model to determine each participant’s change in the two Wave V metrics with stimulus SNR. A model with the same structure was also fit for Wave V amplitude normalized to each individual’s amplitude in the clean condition. These slope fits, along with Wave V latency, amplitude and normalized amplitude were tested for a relationship with the behavioral CRM thresholds for peaky speech using Pearson’s *r*.

#### Determining minimum necessary experimental conditions

The slopes of the change in Wave V amplitude and latency as SNR is decreased represent interesting parameters for future studies of individual differences. To expedite such studies, we determined whether those slopes could be accurately determined from only the extreme SNR conditions, as opposed to all four tested here. We first fit a slope–intercept model to the full dataset for each participant (four points). Next, we did the same for only the clean and −6 dB conditions (two points). We then computed the squared correlation (variance explained) between the slopes from the full and reduced datasets across participants, with higher numbers indicating a better match (and thus lesser importance of the inner two SNRs). We note that since the number of points in the reduced set for each participant matches the number of parameters in the model (slope and intercept), the resultant model is equivalent to simply “connecting the dots.” We carried out this process for both Wave V amplitude and latency.

### Software accessibility

The software used to analyze the data and generate the figures that appear in this paper can be accessed at https://github.com/polonenkolab/peaky_snr. All experimental data is uploaded to Openneuro and can be accessed at https://openneuro.org/datasets/ds005408/versions/1.0.0.

## Results

### Speech-in-noise thresholds are the same for unaltered and peaky speech

Before assessing the neurophysiological effects of speech-on-speech masking, it was important to confirm that the perceptual masking effects for peaky speech matched that of the unaltered speech stimuli. Figure 2 shows the thresholds, expressed in decibels TMR, for each participant as well as the average. The mean ± SEM thresholds were 1.20 ± 0.31 and 1.38 ± 0.33 dB for unaltered and peaky speech, respectively. This difference of 0.18 ± 0.02 dB was not significant (paired *t* test; *t*_(24)_ = −0.92; *p *= 0.37).

### ABRs change systematically with worsening SNR

Clear ABRs were present for all SNR conditions (Fig. 3). Even though early ABR Waves I and III were present in most participants (visible at ∼3.5 and 5.5 ms in Fig. 3), they were less distinct than Wave V, which makes identifying trends in small differences across SNR conditions difficult. As such, we focused on SNR-dependent changes to the amplitude and latency of Wave V.

**Figure 4.**
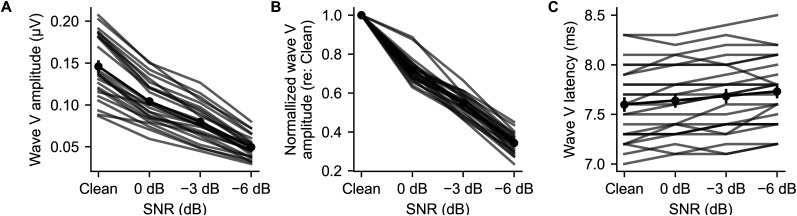
Wave V amplitude [in microvolts (***A***) and normalized (***B***)] and latency (***C***), across SNR conditions. Gray lines show individual participants. Black points and error bars show mean and ±1 SEM.

There was a pronounced and consistent reduction of Wave V amplitude with decreasing SNR for every participant (Fig. 4*A*). The mean amplitude for the clean speech was 0.15 μV, shrinking to 0.10, 0.08, and 0.05 μV for the 0, −3, and −6 dB SNR conditions, respectively. The three oldest participants had three of the four smallest responses of the group, which is consistent with a possible age effect. However, a study specifically investigating age would need to confirm this effect: although there was a range of ages, the distribution was highly skewed and does not lend itself to further analyses (20/25 participants were between 19 and 25 years old; 3 were in their late 30s, and the next oldest was 28 years). An ANOVA following a linear mixed-effect model of Wave V amplitude with SNR and gender as fixed effects and participant as a random effect shows a strong, significant effect of SNR [*F*_(1,23)_ = 371.5; *p *= 1 × 10^−15^; *η_p_*^2^ = 0.94; estimated intercept ± SEM, 0.159 ± 0.008 µV; estimated slope ± SEM, −0.046 ± 0.002 µV/log_2_(# talkers)], as well as gender (main effect, *F*_(1,23)_ = 7.2; *p *= 0.013; *η_p_*^2^ = 0.24; estimated intercept ± SEM change for males, −0.037 ± 0.014; interaction, *F*_(1,23)_ = 8.1; *p *= 0.009; *η_p_*^2^ = 0.26; estimated slope ± SEM change for males, 0.012 ± 0.004). Overall, the Wave V amplitudes for males were smaller in the clean condition and showed a shallower decrease in amplitude with increasing SNR.

Closer examination of the data revealed that Wave V amplitude in the clean condition and its change with SNR were highly related rather than independent metrics—people with larger responses had proportionally steeper slopes. Thus, the data were replotted in Figure 4*B* with Wave V amplitude as a function of SNR normalized to each participant’s amplitude for the clean condition. These normalized Wave V amplitudes showed a strong proportional change with SNR [*F*_(1,23)_ = 1,115.1; *p *< 2 × 10^−16^; *η_p_*^2^ = 0.98; estimated intercept ± SEM, 0.997 ± 0.022 AU; estimated slope ± SEM, −0.286 ± 0.010 AU/log_2_(# talkers)], but gender no longer had an effect (main effect, *F*_(1,23)_ = 0.02; *p *= 0.886; *η_p_*^2^ = 9 × 10^−14^; interaction with SNR, *F*_(1,23)_ = 0.18; *p *= 0.674; *η_p_*^2^ = 7 × 10^−14^). The clean condition was not included in this model due to a violation of homoscedasticity, but very similar numbers are given when the model is run with the clean condition included (i.e., intercept, 0.999; slope, −0.287).

Wave V latency increased with worsening SNR (Fig. 4*C*). The mean latency for clean speech was 7.60 ms, increasing to 7.64, 7.68, and 7.73 ms for 0, −3, and −6 dB SNR. Even though there was more variation across SNR in the individual participant latencies than for amplitudes, all 25 participants showed an overall increase in latency when fit with a line. An ANOVA with the same structure as above shows a significant effect of SNR on Wave V latency [*F*_(1,23)_ = 33.84; *p *= 6 × 10^−6^; *η_p_*^2^ = 0.60; estimated intercept ± SEM, 7.458 ± 0.083 ms; estimated slope ± SEM, 0.061 ± 0.011 ms/log_2_ (# talkers)] and longer latencies for males (*F*_(1,23)_ = 7.37; *p *= 0.012; *η_p_*^2^ = 0.24; estimated intercept ± SEM change for males, 0.375 ± 0.138 ms) but no interaction between gender and SNR [*F*_(1,23)_ = 0.21; *p *= 0.648; *η_p_*^2^ = 9 × 10^−3^; estimated change in slope ± SEM for males, −0.009 ± 0.019 m ms/log_2_(# talkers)].

### Responses are quick to record across stimulus SNR conditions

**Figure 5.**
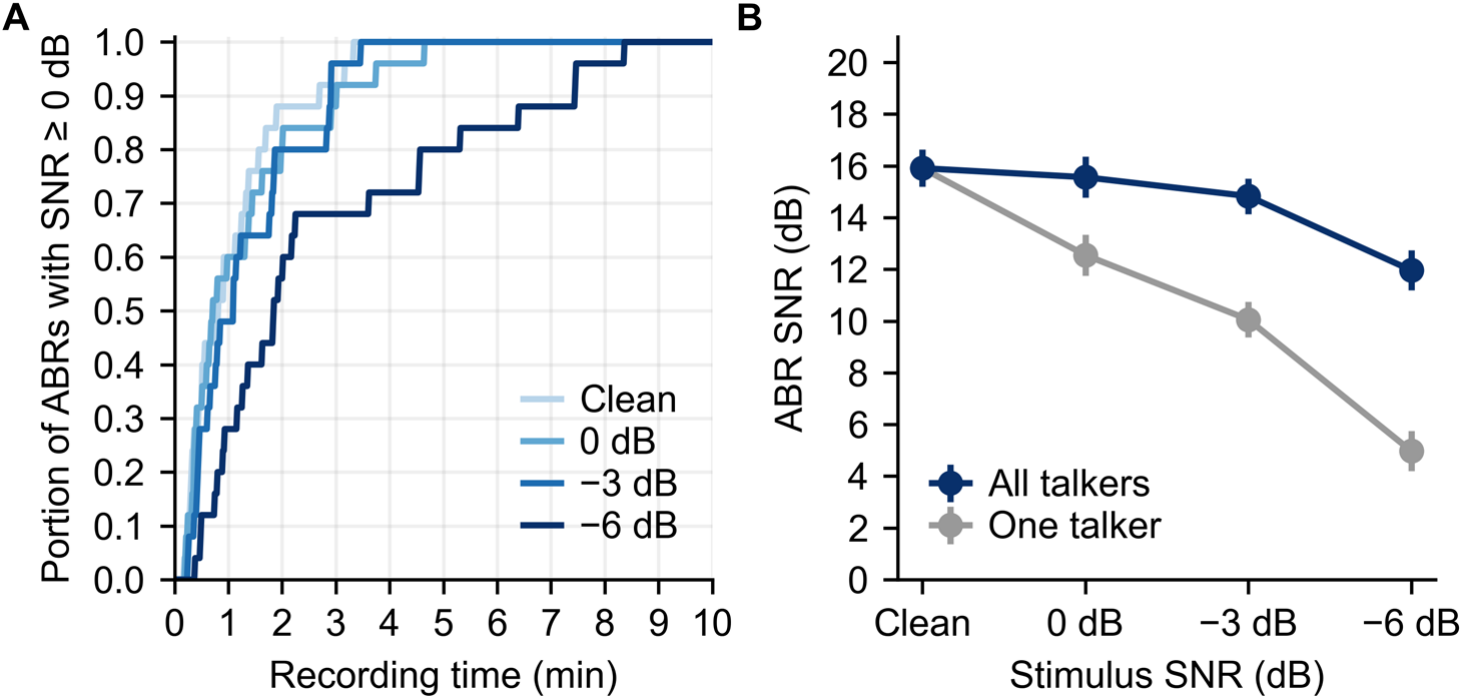
***A***, Cumulative distributions of recording time for each participant's ABR to reach at least 0 dB SNR for each stimulus SNR condition. ***B***, Grand average ABR waveform SNR (computed in the 4–12 ms region) for each stimulus SNR condition using all recorded data (30 min per condition). The dark line indicates using all talkers and averaging (e.g., five talkers in the −6 dB condition), with light gray indicating what the SNR would be if only one talker were considered the target. Error bars show ±1 SEM.

Recording time is a limiting factor in every electrophysiological experiment, so we consider that next. A recording can be considered complete when the quality of the response reaches a certain threshold—in this case, we calculate the point in the acquisition at which the response waveform reaches an SNR of 0 dB, consistent with many prior studies (note that this response SNR is distinct from the speech SNR that comprises this experiment’s primary independent variable). Recording time cumulative distributions are shown in Figure 5*A* for each speech SNR condition. For clean and 0 and −3 dB conditions, 90% of recordings took ∼3 min, and all were under 5 min. For the −6 dB condition, 90 and 100% of recordings were completed in 8 and 9 min, respectively.

The waveform SNR (which is the primary determinant of recording time) is shown in dark line in Figure 5*B*. The average SNRs were 15.8, 15.5, 14.7, and 11.8 for the clean through −6 dB conditions after the entire recording time of 30 min each.

The SNR difference of 4 dB between the two extreme conditions (clean and −6 dB) is smaller than might be expected prima facie given the substantial two-thirds reduction in wave V amplitude between them. The reason for this discrepancy is that, even though the actual recording time is 30 min for both conditions, the duration of speech stimuli presented and used to calculate the responses was not. In the clean condition (one talker), there were 30 min of speech presented in the 30 min recording. For the −6 dB condition, there were five concurrent talkers, meaning that 150 min of speech stimulus were presented in the 30 min recording. A fivefold increase in data leads to a noise reduction (and SNR improvement) of 7 dB. The 0 and −3 dB stimulus conditions benefit similarly from the recording scheme, offering waveform noise reductions of 3 and 5 dB. The light gray line in Figure 5*B* shows what the SNR would be if only one masked talker was presented at a time.

We used four SNR conditions here so that smooth changes in ABR morphologies could be observed, but recording time could be further reduced by running a subset of conditions. To determine such a scheme’s viability, we determined how well the change in Wave V amplitude and latency across SNR could be predicted from only the extreme SNRs (clean and −6  dB) instead of all four datapoints for each participant (see Materials and Methods for details). For Wave V amplitude, the variance explained between the full and reduced datasets was 99.7%. For latency it was 97%. Both of these are strikingly high, indicating that the intermediate SNRs offer essentially no additional information for computing the slopes that is not already present in the extreme conditions. When we follow the same procedure but keep only the inner SNRs instead of the extremes, the variance explained by the reduced model is only 36 and 8% for Wave V amplitude and latency, respectively, indicating that the prior numbers are not artificially high as a result of including common datapoints in both models. Thus, for experiments (or diagnostics) where the variation in Wave V amplitude and latency across stimulus SNR is of interest, two conditions may provide essentially the same information that four do.

### Exploratory analyses find no correlation between ABR and behavioral speech-in-noise thresholds

**Figure 6.**
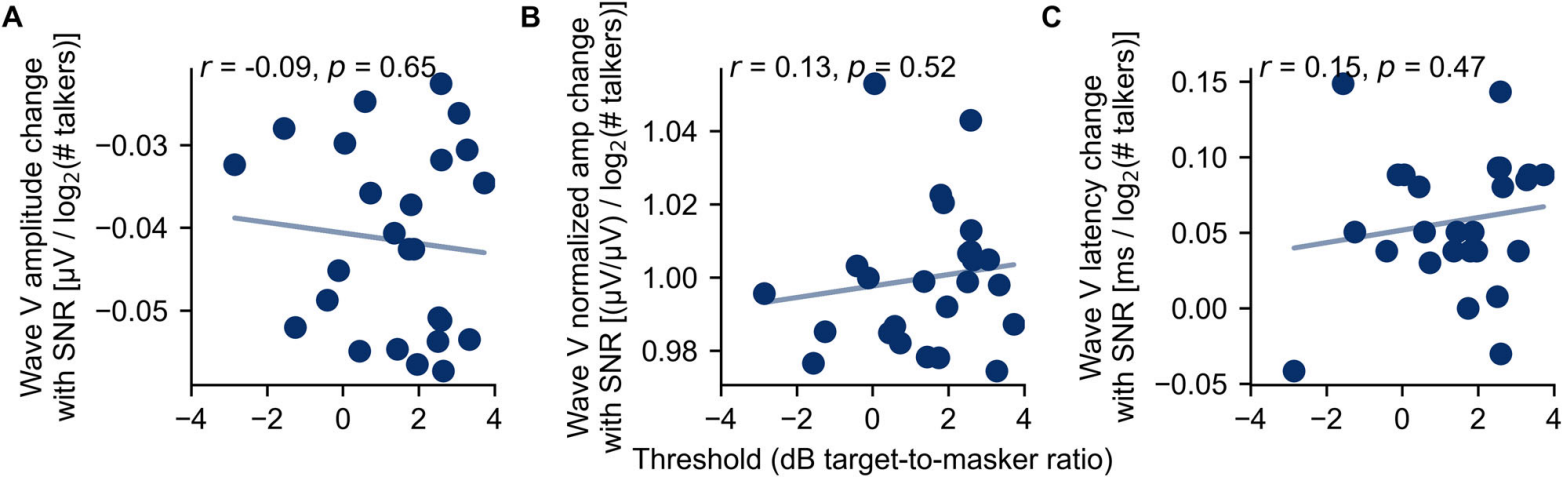
The relationship of speech-in-noise thresholds with the slope of the Wave V amplitude change [in microvolts (***A***) and normalized (***B***)] and latency change ***C***, across SNR. Individual participants are plotted as points along with the best linear fit. There was no significant correlation between any Wave V parameter and speech-in-noise perception.

The relationship between ABR changes with stimulus SNR and perception are of great interest for future work, but were not a focus of this study. Still, because we tested speech reception thresholds before the ABR experiment, we performed an exploratory analysis to see if they were related. The results are plotted in Figure 6. The correlation between Wave V amplitude change (computed as the slope term of a first-order fit of each subject’s data) and speech reception threshold was not significant when expressed in microvolts (*r *= −0.09; *p *= 0.65) or when normalized to each subject’s clean Wave V amplitude (*r = *0.13; *p *= 0.52). The correlation between Wave V latency change and speech reception threshold was also not significant (*r *= 0.15; *p *= 0.47). It is important to note that the participants tested in this experiment were young listeners with normal hearing thresholds, and we further have no reason to believe they struggle with listening in noise. There is thus very little variance to explain. Future studies will require recruitment of participants that span the range of whatever aspect of auditory function is of interest.

## Discussion

This study aimed to understand how the subcortical response to continuous speech changes under energetic masking by other speech. This is an important question to address because understanding speech in noisy scenarios is both a common and frustrating experience for many people. While behavioral studies of masking are plentiful, physiological studies aimed at the subcortex are far fewer in number and have faced limitations. Several studies have measured the frequency-following response to a repeated syllable presented in babble noise (Wong et al., 2007). Those responses, however, are generated by an unknown mixture of sources, including both subcortical and cortical areas (Coffey et al., 2016, 2017, 2019). Other studies have measured a canonical ABR with clear generators but have used artificial stimuli such as clicks that are far removed from natural speech (Mehraei et al., 2016). In this study, we utilized the peaky speech TRF paradigm (Polonenko and Maddox, 2021a), which allows clear ABRs to be measured from naturalistic speech stimuli (here, audiobooks).

### Effects of speech masking on subcortical continuous speech encoding

We observed two significant effects of SNR on the encoding of individual speech streams: the Wave V component of the ABR decreases in amplitude and increases in latency as the number of competing streams increases. These changes were quantified by extracting amplitude and latency parameters of individual response waveforms (Fig. 4, with individual responses shown in Extended Data Fig. 3-2) as well as the grand averaged waveforms (Fig. 3). These changes in Wave V are similar to a recent study that examined the effect of the sound level of a single speech stream using analysis methods much like ours (Kulasingham et al., 2024b). Thus, two distinct factors that can make speech more difficult to understand (either reducing its energy or increasing the energy of competing sounds, thereby decreasing the SNR) both exert similar effects on the subcortical response waveform.

The focus on ABR Wave V here (and in other papers) is mostly one of convenience: Wave V is a large and distinct peak in each participant’s response. The ABR waveform has additional components originating from the ascending pathway’s earlier neurogenerators (Wave I, the small bump at ∼3 ms; Wave III, the bump at ∼6 ms on the rise to the Wave V peak) that can be seen in the grand average waveforms. Wave III, which is generated by the cochlear nucleus (Burkard et al., 2006 p.5), seems to show a similar amplitude change as Wave V without much change in latency, but the component was not robust enough for trends to be analyzed at the level of single-participant waveforms. Wave I, generated by the auditory nerve, was similarly too small for detailed analysis, even though it was present in the grand averages. The compound action potential (CAP) is a response with the same generator as Wave I that is measured with an electrode placed on the eardrum (Simpson et al., 2020). It is substantially larger than Wave I with much better SNR, but the placement of the electrode adds complication to running the experiment and was not pursued here.

There are many possible stimulus regressors for computing the subcortical TRF with different strengths and weaknesses (Kulasingham et al., 2024a). Recent work from our lab directly compared the peaky speech paradigm’s glottal pulse regressor to a different regressor based on the predicted firing rate of an auditory nerve model and found that the latter yields slightly higher SNR (Shan and Maddox, 2024). We opted to use the glottal pulses here because, unlike the auditory nerve model, it provides amplitudes in physical units (µV) and the latencies of the responses do not require adjustment to compensate for the estimated model delay (Shan et al., 2024). Responses computed with the auditory nerve model regressor are included in Extended Data Figures 3-3 and 3-4.

10.1523/ENEURO.0561-24.2025.f3-3 Figure 3-3 Grand average waveforms generated from the auditory nerve model regressor (Shan et al., 2024). Plotted as in Figure 3. Download Figure 3-3, TIF file.

10.1523/ENEURO.0561-24.2025.f3-4 Figure 3-4 Individual participants’ responses for each SNR condition, using the auditory nerve model regressor (Shan et al., 2024). Wave V peaks and troughs are marked with dots and exes, respectively. Download Figure 3-4, TIF file.

### Practicality for clinical measurement

In addition to investigating the overall effects of SNR on brainstem responses, we are also interested in adapting this paradigm for potential clinical use. Neuropathy and synaptopathy of the auditory nerve have been hypothesized to play a role in poorer speech-in-noise perception in people with normal audiologic pure-tone thresholds (Bharadwaj et al., 2014). Finding an indicator of disordered neural processing that is reliable across individuals would be highly valuable, though proven difficult so far (Prendergast et al., 2015; Plack et al., 2016). Wave I (which was present in our data but too small to show trends) is often the response of interest, since it indexes auditory nerve activity (Grant et al., 2020). To evoke robust Wave I responses, measurements are typically done with very high-level clicks and sometimes a horizontal electrode montage that references one ear to the other. The present study eschewed clicks for natural stimuli, and the horizontal montage could not be used because both ears received identical signals, meaning the responses would cancel out. Using an eardrum electrode to measure the auditory nerve CAP allows lower-level stimuli to be used (Simpson et al., 2020). An ecologically relevant paradigm that measures the CAP evoked by a speech mixture at a conversational listening level with an eardrum electrode may provide a more informative clinical measure of auditory nerve encoding.

In addition to the effects that masking has on subcortical speech responses, we also assessed how quickly those effects could be estimated. Responses for the clean and 0 and −3 dB conditions reached the criterion SNR of 0 dB in under 5 min for all participants, with the −6 dB condition reaching criterion in under 10 min for all participants (Fig. 5). The median time to reach criterion was under 2 min for all SNR conditions. Since the Wave V amplitude and latency showed consistent changes as SNR decreased, a clinical test based on the slope of those changes could be sped up by reducing the number of responses measured. We found that using only the clean and −6 dB conditions to compute the slopes provided essentially all the information present in the complete dataset. With only two conditions needed, a simple comparison between the two conditions (e.g., the ratio between the wave V size between the two conditions) replaces computing the slope, leading to metrics that are quick to measure and simple to calculate.

We also performed a behavioral study of speech recognition thresholds in noise. Its primary purpose was to confirm peaky speech showed the same intelligibility as unaltered natural speech (which it did; Fig. 2), but it also allowed an initial exploratory analysis of whether the SNR-driven changes to the ABR were correlated with behavioral thresholds. That analysis showed no relationship between Wave V amplitude, normalized amplitude, or latency changes with the behavioral results. We caution, however, that this was really an “analysis of convenience.” Participants were all young with normal hearing and were not recruited based on reported listening difficulties or a history of noise exposure, so there was very little variance to explain in the first place. Future studies could focus on people with hearing loss, people with normal thresholds who report listening challenges, or people with a broader range of ages. Even if in those populations, too, no correlations were observed across individual participants, determining the overall effects of SNR on subcortical speech coding in those groups would be novel and interesting to compare with the present paper’s findings.

### Why cortical responses were not studied here

TRFs are frequently used to assess sound encoding in the cortex and can be informative about responses driven by basic acoustics or more complicated aspects of an input stimulus such as phonetic features (Di Liberto et al., 2023) or semantic surprisal (Broderick et al., 2018). The focus of the current investigation was the effect of masking on subcortical encoding, but we have previously shown that the same TRF methods used here will also provide cortical responses, simply by extending the end of the analysis window later and using different off-line filter parameters. We did not analyze them here because several uncontrolled experimental factors could have affected cortical responses. First, participant arousal state was not controlled. Some participants were alert, while others may have dozed off. Sleep has a suppressive effect on cortical evoked potentials (Deiber et al., 1989), but does not appreciably affect subcortical responses (Campbell and Bartoli, 1986)—in fact, a sleeping patient is ideal when subcortical responses are used in a diagnostic ABR exam. Second, even though participants were given the option of reading or watching a subtitled movie, we cannot be certain that they did not decide to listen to excerpts of specific stories (or focus on specific talkers) when they were part of a presented stimulus. Even though that was unlikely given the stimulus randomization, directed attention strongly affects cortical responses (O’Sullivan et al., 2014), while its effect on subcortical responses is contested (Varghese et al., 2015) and generally small, even in studies that report it. Interestingly, prior work has shown that the encoding of both the attended and unattended speech streams are relatively stable (with the attended stimulus showing stronger responses) across a wide range of target-to-masker level ratios (Ding and Simon, 2012). Such a study demonstrates the importance of explicitly controlling for attention when investigating cortical encoding of multiple sound sources.

## Synthesis

Reviewing Editor: Christine Portfors, Washington State University Decisions are customarily a result of the Reviewing Editor and the peer reviewers coming together and discussing their recommendations until a consensus is reached. When revisions are invited, a fact-based synthesis statement explaining their decision and outlining what is needed to prepare a revision will be listed below. The following reviewer(s) agreed to reveal their identity: Chengjie Huang.

These results are consistent with the predictions made in the literature, but further advances the field by establishing an analytical approach to evaluating neurophysiological methods, which are few and far between in past studies.

There are a few minor questions and comments which can enhance the paper by clarifying some of the findings. They are listed below:

1) Demographics: There were 16 female and 9 males in the study. Was there a sex difference effect? Furthermore, the ages ranged from 19-37 years with a mean that was on the lower end of the age range. Was there potentially an aging effect given that late 30s could potentially be classified as "middle-aged" where there has been evidence in the literature where normal hearing begins to deteriorate with speech-in-noise listening?

2) ABR Analysis: It is intriguing that ABR Wave I was barely present in the recordings given that these are young normal-hearing listeners. Using your ABR electrode configuration, is there a possibility to use the horizontal electrodes (left active, right reference or vice versa) to acquire a better recording of Wave I? If this analysis is possible, Wave I data would reveal potential differences across SNRs and further strengthen a hypothesis of synaptopathy using Wave V/I ratios. Perhaps expanding on this in the discussion is required if the analysis is not possible with the electrode configuration.

3) The individual latency data is not as consistent as the amplitude data, with some individuals even decreasing latency after a specific SNR. Is there a component of listening fatigue during the ABR recordings which could attribute to this? In other words, due to the offer of watching a subtitled movie, could there potentially be an effect of attention-dependent listening?

4) The reasoning for why there was not a significant correlation between neurophysiology and behavior was interesting. It is unclear to me why keyword identification in multi-talker sentences was not used for the behavioral component (i.e., using the same stimulus in both behavior and ABR components)? However, the explanation for the dataset is acceptable and it would be worthwhile to explore an aging effect from what I mentioned in Comment 1. This should be mentioned in the discussion as it is highly relevant to its practicality as a clinical measurement.

## Author Response

Response to Reviewers Comment:

These results are consistent with the predictions made in the literature, but further advances the field by establishing an analytical approach to evaluating neurophysiological methods, which are few and far between in past studies.

There are a few minor questions and comments which can enhance the paper by clarifying some of the findings. They are listed below:

Response:

We thank the reviewers for their insightful comments and suggestions, which we have addressed below and in the relevant sections of the manuscript (line numbers also given below). We appreciate the opportunity to revise our manuscript.

Comment:

1) Demographics: There were 16 female and 9 males in the study. Was there a sex difference effect? Furthermore, the ages ranged from 19-37 years with a mean that was on the lower end of the age range. Was there potentially an aging effect given that late 30s could potentially be classified as "middle-aged" where there has been evidence in the literature where normal hearing begins to deteriorate with speech-in-noise listening? Response:

Great question. We did some additional stats modeling and found that sex does affect wave V amplitude (females have larger responses), consistent with previous ABR literature. We have added this to the results (lines 252-257). We did have a range of ages, but the distribution is highly skewed and does not lend itself to further analyses (20 / 25 subjects were between 19 and 25 years old; three were in their late 30s and the next oldest was 28). We now note in the paper (lines 245-250) that the three oldest subjects had three of the four smallest responses, which is consistent with a possible age effect, but a study specifically investigating this would need to confirm it.

As a result of this comment, we also realized that wave V amplitude and its slope were highly related, rather than independent metrics-people with bigger responses had larger slopes. This revealed a proportionality to the change in wave V amplitude with decreasing SNR. As a result, we have added a panel to figure 4 that shows wave V amplitude as a function of SNR normalized to each subject's amplitude for the clean condition and added the relevant text to the methods section (lines 206-211) and results section (lines 258-267). We believe this would be a more informative clinical measure, since random factors such as skull thickness or variation in generators' orientations across people can introduce proportional changes in measured neural responses that would also affect their slope without normalization. We mention this now in the relevant discussion section (lines 395-397).

There was an effect of sex on wave V latency but not its slope with decreasing SNR. This has been added to the results (lines 272-277).

Comment:

2) ABR Analysis: It is intriguing that ABR Wave I was barely present in the recordings given that these are young normal-hearing listeners. Using your ABR electrode configuration, is there a possibility to use the horizontal electrodes (left active, right reference or vice versa) to acquire a better recording of Wave I? If this analysis is possible, Wave I data would reveal potential differences across SNRs and further strengthen a hypothesis of synaptopathy using Wave V/I ratios. Perhaps expanding on this in the discussion is required if the analysis is not possible with the electrode configuration.

Response:

We wish we could do this, but our stimulus presentation paradigm disallows it. We presented stimuli diotically, which means that both auditory nerves are responding to the same stimuli (in roughly the same way). This means a left-right montage will provide essentially no response because it subtracts out.

In future studies, the auditory nerve could be better assessed by using tiptrodes or even an electrode on the eardrum, such as the one developed in Skyler Jennings's lab.

We have edited the discussion to reflect these points (line numbers 379-386).

Comment:

3) The individual latency data is not as consistent as the amplitude data, with some individuals even decreasing latency after a specific SNR. Is there a component of listening fatigue during the ABR recordings which could attribute to this? In other words, due to the offer of watching a subtitled movie, could there potentially be an effect of attention-dependent listening? Response:

We think the relatively lower reliability of the latency compared to the amplitude is just driven by size of the change over the different SNR conditions. The mean difference between the Clean and −6 dB conditions was only 0.13 ms. In an individual subject, the smallest difference we could detect would be 0.1 ms, since we recorded at a sampling rate of 10 kHz. This is starkly different from the amplitude, which changes by a factor of 3 between the SNR extremes.

We are pretty confident that neither fatigue nor attention to the movie are playing a role here. First, because much bigger state differences have minimal effect on the ABR (sleep vs wake, for instance). Second, another study we have currently under review comprises a set of three experiments using similar speech-on-speech material over dozens of subjects, all providing clear evidence that attention (in this case, actively attending to one of two competing stimuli) has no impact on subcortical encoding.

Comment:

4) The reasoning for why there was not a significant correlation between neurophysiology and behavior was interesting. It is unclear to me why keyword identification in multi-talker sentences was not used for the behavioral component (i.e., using the same stimulus in both behavior and ABR components)? However, the explanation for the dataset is acceptable and it would be worthwhile to explore an aging effect from what I mentioned in Comment 1. This should be mentioned in the discussion as it is highly relevant to its practicality as a clinical measurement.

Response:

The focus of the study is on (passive) encoding measured with EEG. Using the coordinate response measure (CRM) task allowed us to quickly assess speech-in-noise performance in order to show that peaky speech was as intelligible as natural speech. An open set keyword task (despite its benefits) would have been slower and reduced the time we had to record EEG. We do note, though, that the maskers in the behavioral experiment were drawn from the same audiobook stimuli that we used in the EEG recording (line 95).

As we describe above, we have now added some discussion that there could be an aging effect, but we need more power (results section, lines 245-250). We added populations with a broader age range to the discussion about future studies in different populations (lines 406-407).
