# Supplementary figures and images for: The Effect of Speech Masking on the Human Subcortical Response to Continuous Speech
Source: eNeuro. 2025 Apr 1;12(4):ENEURO.0561-24.2025. doi: 10.1523/ENEURO.0561-24.2025 (PMC11974362; doi:10.1523/ENEURO.0561-24.2025)

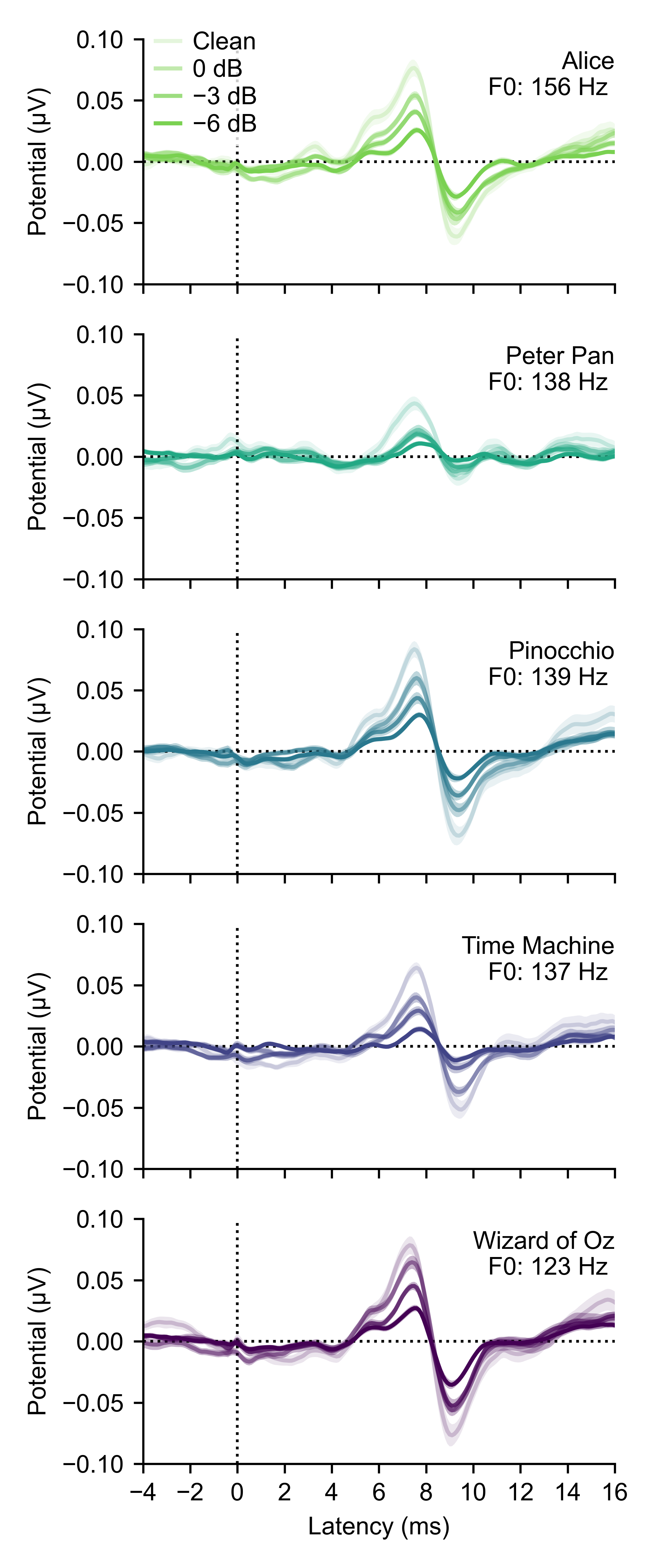

Supplement: Figure 3-1 — Grand average waveforms in response to each of the five stories. Plotted as in Figure 3. Download Figure 3-1, TIF file. [file eneuro-12-ENEURO.0561-24.2025-s003.tif]

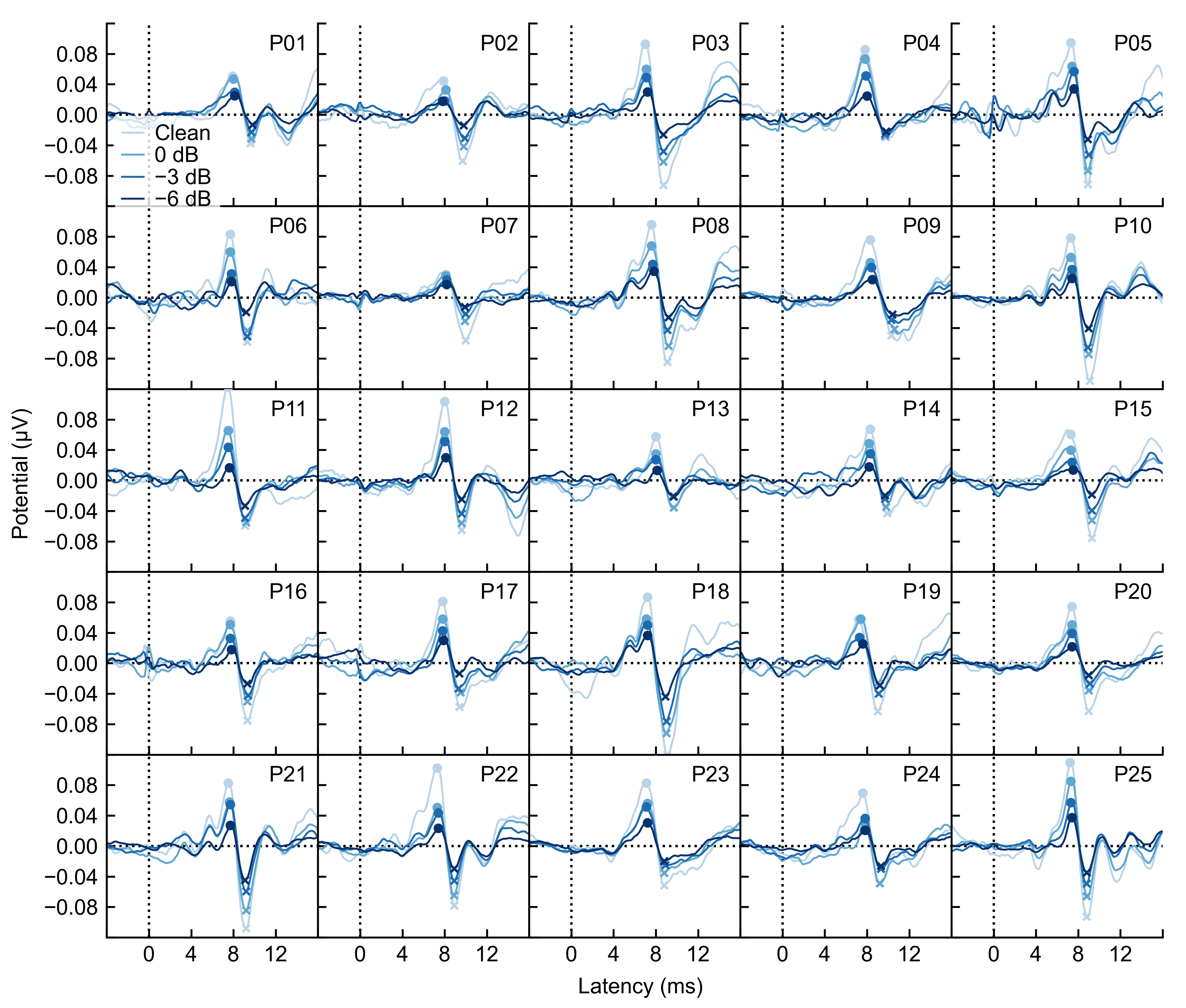

Supplement: Figure 3-2 — Individual participants’ responses for each SNR condition. Wave V peaks and troughs are marked with dots and exes, respectively. Download Figure 3-2, TIF file. [file eneuro-12-ENEURO.0561-24.2025-s004.tif]

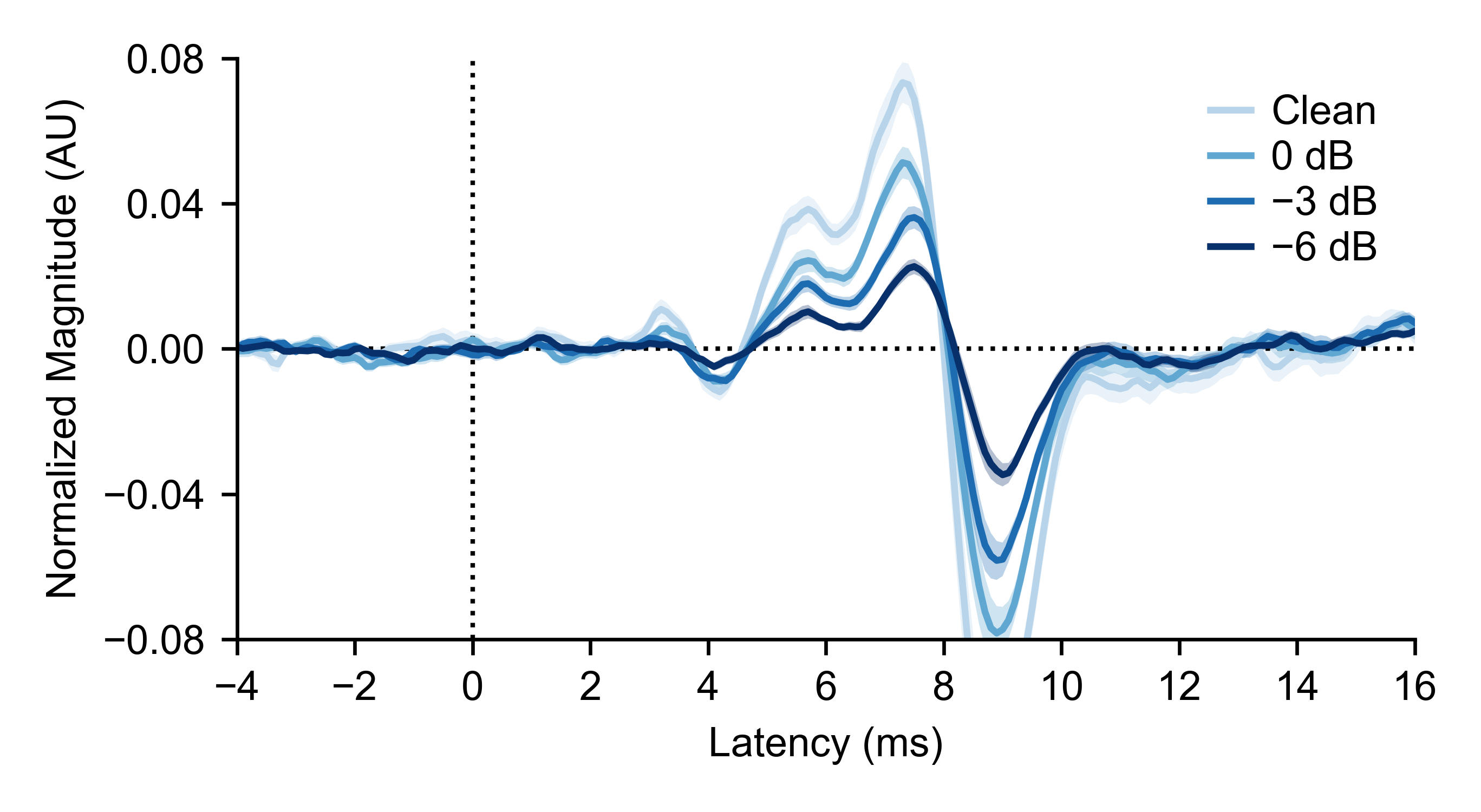

Supplement: Figure 3-3 — Grand average waveforms generated from the auditory nerve model regressor (Shan et al., 2024). Plotted as in Figure 3. Download Figure 3-3, TIF file. [file eneuro-12-ENEURO.0561-24.2025-s005.tif]

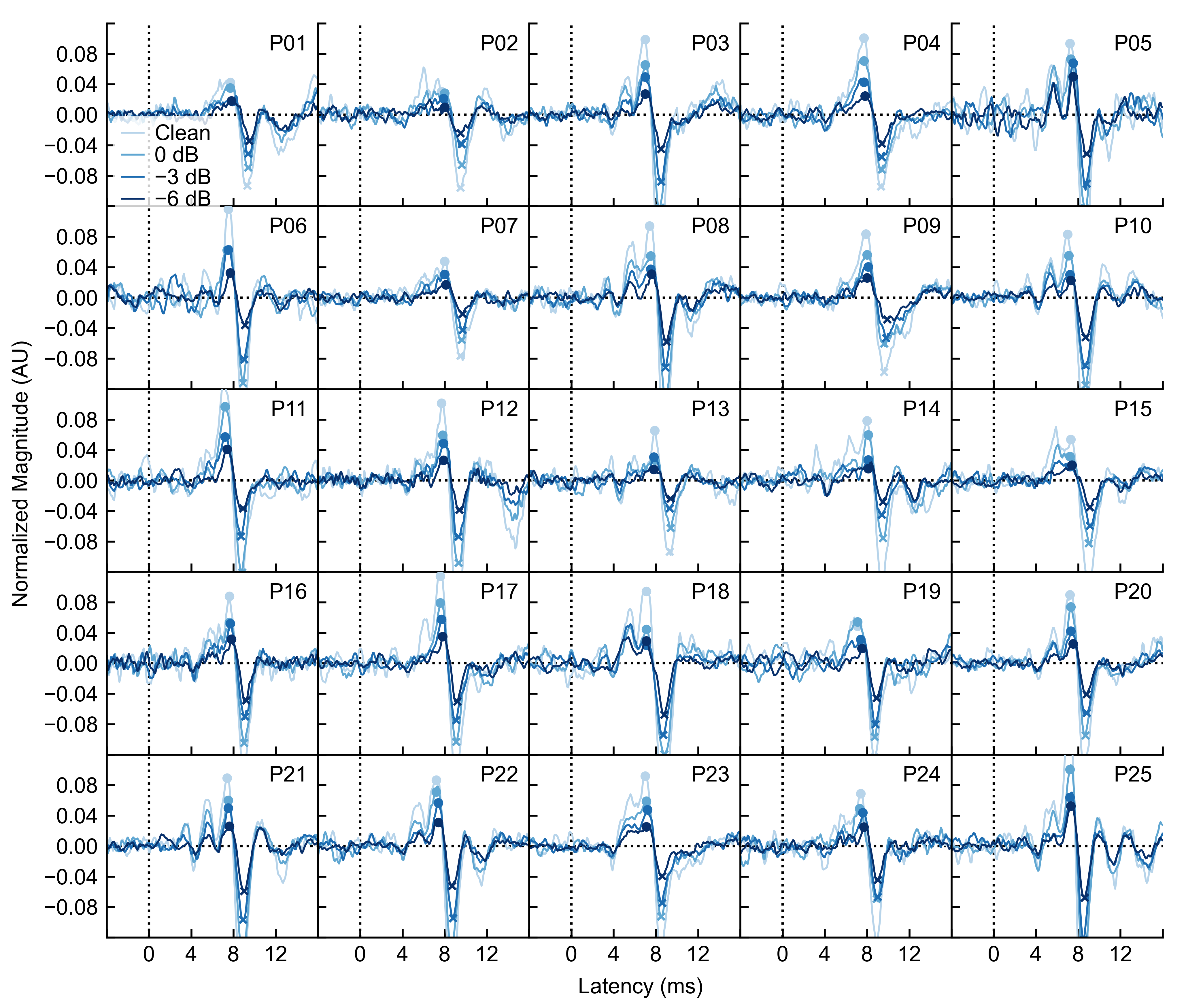

Supplement: Figure 3-4 — Individual participants’ responses for each SNR condition, using the auditory nerve model regressor (Shan et al., 2024). Wave V peaks and troughs are marked with dots and exes, respectively. Download Figure 3-4, TIF file. [file eneuro-12-ENEURO.0561-24.2025-s006.tif]
